# Machine Learning-Based Kinetic Modeling of the CO_2_ Methanation Reaction over an Industrial Catalyst

**DOI:** 10.1021/acs.iecr.5c02947

**Published:** 2025-10-04

**Authors:** Hugo Pétremand, Julia Witte, Oliver Kröcher, Emanuele Moioli

**Affiliations:** † PSI Center for Energy and Environmental Sciences, Paul Scherrer Institute, 5232 Villigen, Switzerland; ‡ Institute of Chemical Sciences and Engineering, Ecole Polytechnique de Lausanne, 1015 Lausanne, Switzerland; § 707342 Kanadevia Inova AG, Hardturmstrasse 127, 8005 Zürich, Switzerland; ∥ Dipartimento di Chimica, Materiali e Ingegneria Chimica ‘Giulio Natta’, Politecnico di Milano, 20133 Milano, Italy

## Abstract

In industrial practice, it is common to use commercially available catalysts to facilitate chemical reactions. Unfortunately, due to the confidential composition and complex properties of the catalysts, a precise understanding of the reaction mechanism and intermediates is often difficult to obtain. This complicates the development of robust kinetic models, delaying an effective reactor design. This raises the question of whether machine learning (ML)-based regressions can reliably describe kinetics without requiring detailed reaction mechanisms. In this work, this research question was addressed by performing kinetic experiments on a commercial Ni/ZrO_2_-based CO_2_ methanation catalyst (Himetz by Kanadevia Corporation) in an isothermal, fixed bed reactor. A 216-point data set was thus obtained at various temperatures (220–300 °C), partial pressures (0.8–3 bar) and gas hourly space velocities (50,000–700,000 h^–1^) combinations. The data set was employed to perform several ML-based regressions, and to compare them in terms of adequacy of the fit, according to root mean squared error (RMSE). The two best performing ML-based models, Gaussian Process Regression and 1-layer neural network were selected and assessed against two standard kinetic modeling approaches: power law and Langmuir–Hinshelwood-Hougen-Watson (LHHW). The ML-based models outperformed the power law according to RMSE and were comparable to the LHHW model to describe the kinetic regime of the catalyst. When implemented in a reactor model, the ML-based regressions also accurately predicted methane yield with results comparable to the state-of-the-art LHHW model and even outperformed the LHHW model by addressing nonideal behavior in a nonisothermal reactor. This showed that ML-based kinetics are promising in situations where little mechanistic information is available, generating models that can be employed for reactor design purposes.

## Graphical abstract

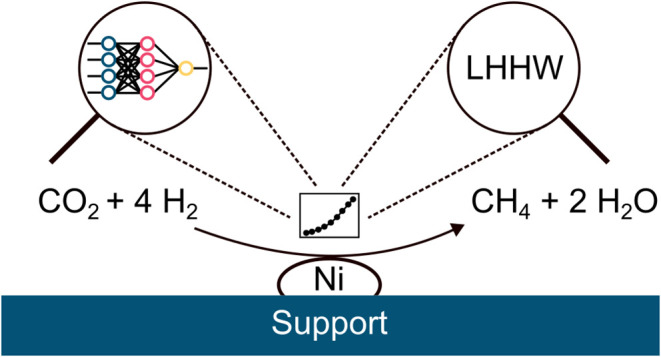

## 1 Introduction

Kinetic modeling is an essential element in chemical reaction engineering, as kinetic models are the basis for reactor simulation, optimization and control.^1^ Over the last decades, several research efforts have been made to obtain ever more precise kinetic models, often based on detailed mechanistic data.^2^ In this sense, the development of *operando* analytical methods has been helpful for the development of detailed kinetic models, as it allows measuring the formation and decomposition of surface species during the chemical reaction, which is the required for the determination of reaction mechanisms and the definition of the number of parameters to regress.^3^ However, to reach this level of detail, time-consuming investigations are required. The length of the kinetic modeling phase risks to delay the development of effective reactor designs and bringing new technologies to the market, as reliable kinetic models are the essential cornerstone of development and scale up.^4–6^

The difficulty in deriving detailed reaction mechanisms, due to several reasons, such as nondisclosure agreements (which limit the allowed analyses), reduced time for experiments (due to budget limitations) or difficulty in defining the reaction mechanisms themselves, has often led research scholars to develop surrogate models. Despite the lower precision of these models relative to mechanism-based models, they can be helpful for reactor design. The most widely used surrogate model type is the power law (PL).^7^ However, the capacity of surrogate models to fit experimental data and to extrapolate reaction rates tends to be limited due to their nonadherence to the actual reaction mechanism. PLs reduce the complexity of the description of catalytic reaction mechanisms to a logarithmic correlation, which holds only for a limited set of parameters. Another widely employed option for the description of catalytic reaction involves the adaptation of kinetic models available in literature by using appropriate correction factors. However, such models are rarely available when working with specialized catalysts of nonstandard compositions and the adaptation to new catalysts is often challenging.

In recent years, with the advances in machine learning (ML), the possibility of applying artificial intelligence (AI), using machine-learning-based regressions for surrogate kinetic modeling has become a new topic of research.^8,9^ For reactor design purposes, the main goal is to obtain a reasonable prediction of the reaction rate over a large set of parameters. It is therefore feasible to use ML-based approaches that hint to physical correlations without involving real descriptions of reaction mechanisms. The current availability of cheap computational power may push the development of surrogate ML driven models aimed at reproducing kinetic correlations over a wide range of parameters. Hence, various types of kinetic models were suggested, ranging from black-box-style deep neural networks (NN)^10^ to physically informed kinetic expressions.^11–14^ Recently, it was demonstrated that artificial neural networks (ANNs) can effectively predict microkinetics when the right physical constraints are applied.^15^ This work aims to systematically extend the knowledge on the suitability of ML-based methods for deriving kinetic expressions, based on new experimental data obtained from a commercial CO_2_ methanation catalyst. The experimental data were obtained in a simple lab reactor, to generate a kinetic data set that can be retrieved with a relatively low experimental effort and without using specialized equipment. The kinetic models were regressed splitting the data set into training and test sets using a 5-fold cross-validation scheme. This means that the data set is split into five equally large parts, 4 used for regression and 1 for testing. The procedure is repeated 5 times, each time with a different fold used as the test set, to ensure the development of a statistically relevant model.

For the development of kinetic models, a wide array of available ML-based regression models, including random forests, ANNs and Gaussian process regressions (GPRs) were analyzed and compared to a standard PL surrogate model. Additionally, the most promising ML-based regression was compared to an adapted Langmuir–Hinshelwood-Hougen-Watson (LHHW) model obtained from literature.^16^ The final aim of this work was to determine whether ML-based kinetic models can describe the effect of several parameters in a wide operating range, and hence if they can be employed in industrial reactor design with an accuracy comparable to the state-of-the-art mechanistic models.

CO_2_ methanation, or the Sabatier reaction^17^ was used as reference reaction. It follows the stoichiometry in eq 1

1 $$CO_{2}+4H_{2}⇄CH_{4}+2H_{2}O$$

and it was identified as an ideal reference reaction for this study due to several reasons. Primarily, the industrial catalyst employed in this study is highly selective toward methane, hence allowing the description of the system with a single rate equation. Secondarily, several catalysts were tested for this reaction and several kinetic models are available in literature,^16^ especially for Ni-based systems (such as Ni/Al_2_O_3_ ^18^ and Ni/SiO_2_ ^2,19–21^ ). Additionally, the rapid development of renewable energy production and decrease in production costs^22^ have greatly renewed interest in said reaction as it produces a common fuel (methane) from a typical energy storage molecule (hydrogen). As renewable energy commonly originates from intermittent sources, energy storage is essential to avoid curtailment and therefore the use of H_2_-derived methane as a sustainable energy carrier has gained interest. The benefits of methane used in a power-to-gas scheme include its energy density and ease of transport via the preexisting gas network,^23,24^ as well as the upcycling of CO_2_, which will be essential for the energy transition. This concept will however require process intensification and miniaturization of reactors to be used effectively, hence the need for adequate kinetic models is urgent.^25^

The study was structured into model development, evaluation, and application to industrially relevant cases. The first section focuses on the description of the kinetic models developed, showing the difference in prediction performance of the various methods. Afterward, the performance in data prediction of the various regression types is evaluated by decreasing the number of data points of the training data set, evaluating how the various methods react to this change. The best kinetics derived are then implemented in black-box, isothermal and nonisothermal reactor models and their performance in predicting conversion profiles was compared with the state-of-the-art LHHW model. In this way, it is possible to understand the potential and limitations of ML-based models compared to the traditional kinetic modeling approach according to the desired final modeling scope.

## 2 Materials and Methods

### 2.1 Experimental Section

The kinetic experiments were performed in a bench scale fixed-bed reactor, as shown in Figure S1. The reactor consists of a metal jacketed tube (ID = 21 mm, *L* = 12 cm) with forced air temperature regulation (200 L·h^–1^, *T* = 15–350 °C), and a glass frit to distribute the gaseous flow and support the packing material. The catalyst used was Ni-based, supported over ZrO_2_. The catalyst (HiMetz) was produced and supplied by Kanadevia Corporation (Japan) in cylindrical pellets of 3 mm × 3.6 mm dimensions. The catalyst was ground and sieved to obtain particles of 1 mm size, to avoid the insurgence of intraphase heat and mass transfer limitations. The absence of mass transfer limitations was verified experimentally by varying the catalyst particle size and theoretically by applying Mears’ correlations.^26^ The packing consisted of a 5 cm layer of glass beads, followed by a 6 cm catalytic zone, composed of the catalyst (*D* = ∼1 mm, 0.720 g) dispersed in glass beads (*D* = 1 mm). The catalyst dilution was necessary to avoid the insurgence of a temperature hotspot and to obtain an isothermal bed. The first layer of glass beads was used to ensure preheating of the gas stream to the desired temperature when operating at high gas hourly space velocities (GHSV). Temperatures were measured throughout the setup with thermocouples installed under and above the frit as well as in the catalytic bed, via the use of a height adjustable probe in the middle of the reactor. Said probe consists of 5 thermocouples set 2 cm apart in a thermal well. In the kinetic experiments, thermocouples were positioned at 1, 3, and 5 cm height above the frit (position 0) in the catalyst bed, and 1 cm below and above the catalyst bed. Gases (Linde: H_2_ 4.5, CO_2_ 5.3, Ar 5.0, and Messer: N_2_ 5.0) were fed by means of calibrated electronic mass flow controllers. The reactive gases could either be sent to the reactor or a bypass line. The streams from bypass as well as from the reactor outlet were connected to a mass spectrometer (MS) (Pfeiffer Vacuum OmniStar GSD 301 01) for composition analysis. The reactor and bypass gas streams were then combined and passed through a condenser to remove water before passing through a nondispersive infrared (NDIR) sensor, to measure CO, CO_2_ and CH_4_.

Prior to initial kinetic experiments, the catalyst was activated under diluted H_2_ (10% in Ar, 3 bar, 120,000 h^–1^) for ca. 30 min at 230 °C. Upon initial H_2_ addition, a 0.8 °C exotherm was measured alongside methane and water traces, which were used as indicators for activation of the nickel catalyst used. One single catalyst sample was used for all kinetic experiments, as no signs of deactivation were observed over time. This was controlled by measuring the methane yield at a reference point (230 °C, H_2_/CO_2_ = 4.0, 140,000 h^–1^), which was regularly tested to be constant throughout the study. The experimental conditions tested are listed in Table 1. Temperature, GHSV and H_2_/CO_2_ ratio were the main parameters of interest, whereas the pressure was kept constant at 3 bar absolute in the kinetic study, because this is the maximum pressure allowed in the setup. Varying the total pressure would make the kinetic model useful for prediction over a larger set of parameters, but for the scope of this work it was not necessary, as the aim of the study is to verify whether ML-based models can predict the effect of partial pressure change. A plot of the parameter space is shown in Figure S2.

**Table 1:** Experimental Space Explored in the Kinetic Study with Lower and Upper Limits of Temperature, Pressure, GHSV and H_2_/CO_2_ Ratio

parameter	lower bound	upper bound
temperature [°C]	220	300
pressure [bar]	3	3
GHSV [h^–1^]	50,000	700,000
H_2_/CO_2_	0.5	6
Ar [%]	0	75

### 2.2 Modeling

The PL describing the CO_2_ methanation reaction is given in eq 2,^16^ with *k* being the kinetic constant according to the Arrhenius equation. This simple PL model was chosen as the commercial catalyst was 100% selective to methane and did not produce CO. The reaction orders, pre-exponential factor and activation energy were derived by regression from the experimental data set obtained in this study.

2 $$r_{meth}=k\cdot {p_{{CO}_{2}}^{\alpha }p}_{H_{2}}^{\beta }\left(1-\frac{p_{H_{2}O}^{2}p_{CH_{4}}}{p_{H_{2}}^{4}p_{{CO}_{2}}K_{eq}}\right)$$

where *K*_eq_ was approximated using the empirical formula.^16,27^

3 $$K_{\mathrm{eq}}=137\cdot T^{-3.998}\cdot \exp\left(\frac{158.7\;\mathrm{kJ}/\mathrm{mol}}{R\cdot T}\right)$$

The LHHW rate equation shown in eq 4 was obtained from Koschany et al.^16^ The parameters were not derived from the experimental data set but used as provided by the literature source directly. This was done for two reasons: first, the actual mechanism is not known for the catalyst of this study, hence a reparametrization of the adsorption constants would not be conceptually correct. Second, the data set derived in this study would be too small to perform a reliable regression of the 8 parameters of the LHHW model (*k*_0_, *E*_A_, 3 pre-exponential factors for adsorption and 3 adsorption enthalpies). Hence, the only adaptation made to the model was the addition of a correction factor (*f*_corr_) as described in eq 4. In this way, it is possible to keep the formulation of the state-of-the-art mechanistic model, by simply adapting the kinetic constant to the new data.

4 $$r_{\mathrm{meth}}=\frac{f_{c\mathrm{orr}}\cdot k\cdot p_{H_{2}}^{0.5}p_{CO_{2}}^{0.5}\left(1-\frac{p_{CH_{4}}p_{H_{2}O}^{2}}{p_{CO_{2}}p_{H_{2}}^{4}K_{\mathrm{eq}}}\right)}{{\left(1+K_{\mathrm{OH}}\frac{p_{H_{2}O}}{p_{H_{2}}^{0.5}}+K_{H_{2}}\cdot p_{H_{2}}^{0.5}+K_{\mathrm{mix}}\cdot p_{CO_{2}}^{0.5}\right)}^{2}}$$

As for the ML-driven models, the regression learner tool from the Statistics and Machine learning Toolbox from MATLAB R2024a was used. The data sets for the regression were prepared by tabulating the input and output files in an Excel file. The input data selected were the input flow rates (CO_2_, H_2_, Ar) and temperature. The output data were the corresponding experimental CH_4_ yield. CH_4_ yield was chosen as output parameter to have a normalized output function. From the predicted methane yield, the reaction rate was calculated by multiplying for the flow rate of CO_2_ and dividing by the catalyst amount. This is possible because the kinetic data points are obtained in differential conditions. The files were then given as input to the regression learner after randomization. In the regression learner, the various models could be chosen and trained using the supplied data. All available algorithms, ranging from linear and regression trees to ANNs and GPRs were evaluated using the full experimental data set, with most being discarded early due to their inability to include nonlinearity. The models were trained using 5-fold cross-validation and the resulting models were of the form described in eq 5, allowing them to be used as pure kinetic models or supplied to a reactor model. The reaction rate was then calculated by dividing the calculated yield by the catalyst amount (considering that the data points are obtained in differential conditions).

5 $$f\left(\dot{{\mathrm{CO}}_{2}},\dot{H_{2},}\dot{\mathrm{Ar}},T\right)=\mathrm{yield}\left[\%\right]$$

The details of the ML-learning (specified for the full data set case) are as follows:

- Fine tree: the minimum leaf side selected is 4 and no surrogate decision splits were applied. The training time was 2.567 s.
- Neural network: a single layer neural network was used. The NN consists of one single fully connected layer with iteration limit set to 1000. The activation function is the rectified linear unit (ReLU) with a regularization strength of 0.0294. The first layer size is 146. The training time required was 45.687 s.
- Gaussian Process Regression: GPR was performed using a kernel function ‘nonisotropic matern 5/2’. The kernel scale was 970 and the sigma value 4.07. The signal standard deviation was 0.294. The training time was 55.625 s.

#### 2.2.1 Effect of the Parameter Choice on the Regression Performance

The adaptability of the ML-based models was tested by employing both reduced and extended data sets as listed in Table 2. Models were trained on these modified data sets using the same 5-fold cross-validation scheme, allowing for the evaluation of their adaptability to the removal or addition of certain types of data points. To derive the apparent reaction orders of a PL it is necessary to vary the reactant ratio, ideally throughout the temperature range of interest, which leads to additional experiments that are out of the typical range of operation of a reactor. As ML-based regressions do not require explicit understanding of reaction orders, these experiments are potentially unnecessary for their derivation. This could allow decreasing the number of experiments required for obtaining a suitable kinetic model for reactor design purposes. A reduced data set (set 02) was therefore prepared by excluding experiments with variations of the reactant ratio.

**Table 2:** Brief Description of the Datasets Measured and Used for the Model Training

data set	data points	description
01	216	full experimental data set
02	173	set 01 excluding data obtained far from stoichiometry
03	171	set 01 excluding data above 15% methane yield
04	257	set 01 including synthetic thermodynamic equilibrium data points

A second test used a reduced data set with only sub 15% yields (set 03), allowing for comparison of the modeling approaches in a purely differential reactor (far from thermodynamic equilibrium). This allows estimating the impact of the data set size on the resulting model and how adaptable ML-based approaches are to data quantity and quality.

In order to develop an accurate reactor model, it is necessary to accurately describe the behavior of the catalyst at the thermodynamic equilibrium, as this reflects the operating regime of the reactor for high temperatures. PLs and LHHW models intrinsically predict the thermodynamic equilibrium, thanks to the reversible term. However, ML-based models do not explicitly include knowledge of the thermodynamic equilibrium. For this reason, thermodynamic equilibrium data points were added to the training data sets (set 04) to obtain a physically informed ML model with the aim to extend their range of application. The thermodynamic equilibrium data points were simulated at 3 bar, 200–600 °C using Aspen Plus V14, using the Peng–Robinson fluid package, with a “Gibbs” reactor, which minimizes the Gibbs free energy of the system. These data points were supplemented to the training routine as experimental data points obtained at low flow rates (GHSV < 1000 h^–1^). In these conditions, it was supposed that thermodynamic equilibrium would be reached. The thermodynamic equilibrium is hence added to the model as a soft constraint of additional data points that the model needs to predict. Additionally, the thermodynamic equilibrium limit was supplemented as maximum achievable yield in the reactor models, to avoid overestimation of the process yield. This is instead a hard constraint that impedes overestimating the thermodynamic equilibrium conversion at high temperatures. A detailed flowchart of the training and testing routines is provided in Figure S3.

Since the goal of the study is the possible replacement of standard kinetic laws with ML-derived expressions, both types of models were implemented in reactor models. The two reactor models employed here are an isothermal and a nonisothermal CO_2_ methanation reactor model. Already the simulation of an isothermal reactor model greatly increases the complexity of the prediction, as the models needs to describe reaction rates starting from all parameters involved over the axial coordinate of a reactor. The conservation law-based reactor model forces the ML-based model to respect stoichiometry, mass and energy conservation as a hard constraint. The model employed for the simulation of the technical reactor follows the molar and heat balances

6 $$\frac{dF_{i}}{dz}=-\rho_{\mathrm{pb}}\left(1-\varepsilon \right)A\cdot \nu_{i}\cdot r_{\mathrm{meth}}\;\left(m\mathrm{olar balance}\right)$$

7 $$\frac{C_{p,\mathrm{mix}}\left(1-\varepsilon \right)v_{\mathrm{GAS}}dT}{dz}=-\frac{4}{d}U\left(T-\mathrm{Te}\right)-\rho_{\mathrm{pb}}\left(1-\varepsilon \right)\Delta H_{\mathrm{meth}}^{R}r_{\mathrm{meth}}\;\left(h\mathrm{eat balance}\right)$$

For the isothermal model, the Δ*H*_meth_^*R*^ was set as 0. For the reactor geometry, a plate reactor was considered for validation purposes, as experimental data are available for this reactor type, operating with the same commercial catalyst as our study.^28^ The reactor is composed of 5 plates with a distance of 5 cm between each other. The reactor is cooled by boiling water at a temperature of 230 °C, which circulates in the plates. The socket between two plates is filled with the catalyst. The catalyst layer is 1.8 m long. The heat transfer (*U*) toward the cooling medium was calculated as a series of resistances, accounting for the heat transport in the catalytic bed, the resistance of the reactor wall, and the heat transfer in the cooling medium

8 $$\frac{1}{U}=\frac{1}{U_{\mathrm{pb}}}+k_{w}+\frac{1}{U_{\mathrm{bw}}}$$

where pb is the packed bed and bw is the boiling water. The heat transfer coefficient in the reactive medium was calculated as

9 $$U_{\mathrm{pb}}=\mathrm{Nu}\frac{l_{g}}{D}$$

The Nusselt number was calculated as

10 Nu=2+1.1(Pr)1.3(Re)0.6

The heat transfer coefficient of boiling water was considered constant at 2 kWm^–2^K^–1^. The reactor wall is composed of stainless steel and has a thickness of 2 mm. This enables a more thorough evaluation of the kinetic model’s accuracy in predicting reaction rates, which is essential, as the location and intensity of hotspots are critical to plant operability.

## 3 Results and Discussion

The catalysts performance was first tested by varying the temperature under reference conditions. The yield to methane was recorded every 10 °C in the interval 220–300 °C. The determination of the dependence of temperature enables an understanding of the catalyst’s basic reactivity characteristics, which can be incorporated into the kinetic model. Figure 1 shows the results in terms of methane yield as a function of temperature and space velocity. The catalyst is highly active in the CO_2_ methanation reaction and showed a non-negligible methane yield already at 220 °C. With increasing temperature, the methane yield raised quickly, reaching 70% at 300 °C, and 110,000 h^–1^. Hence, low conversion (<50%) and perfectly isothermal reaction conditions were only maintained under 280 °C. Kinetic models could therefore only be parametrized successfully using a data set limited to this temperature range. The experiments did not show the formation of any side product under all reaction conditions. It was hence concluded that the catalyst is 100% selective to methane, and this assumption allowed the kinetic modeling to be reduced to solely the CO_2_ methanation reaction. The calculated activation energy for the methanation reaction was 91.4 kJ/mol, which is in line with literature values of 59–100 kJ/mol for Ni/ZrO_2_,^29–31^ 80–106 kJ/mol for Ni/Al_2_O_3_ ^18,32–34^ and 83 kJ/mol for Ni/Al_2_O_3_ based methanation catalysts.^16^ The reaction orders obtained from regression were 0.123 for CO_2_ and 0.351 for H_2_, also in line with literature data.^16,31,33^

**Figure 1:**
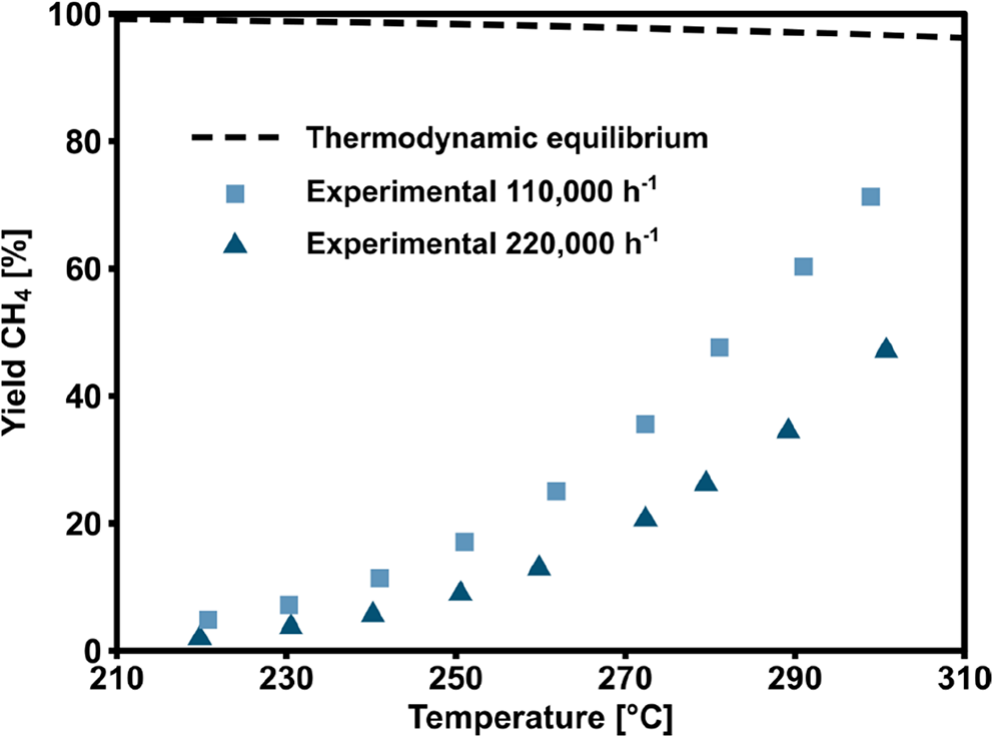
Experimentally obtained temperature dependency of the reaction in the range from 220 to 300 °C, at a GHSV of 110,000 and 220,000 h^–1^.

In comparison to other Ni-based catalysts for CO_2_ methanation described in literature, the commercial catalyst used in this study demonstrated excellent performance, placing it among the most active CO_2_ methanation catalysts reported to date. The trends observed in the experiments and the dependencies from the main parameters precisely followed what was observed in other kinetic modeling studies. For this reason, it was decided to use the same LHHW approach as in the study by Koschany et al.^16^ However, no evidence was found that the reaction follows a similar mechanism on the catalyst of this study compared to the catalyst described by Koschany et al. Hence the use of the rate equation as in eq 4 was solely based on considerations of similitude in the parametric dependencies. Nevertheless, the fitting of this rate equation to the experimental results appeared satisfactory, as it will be shown later.

### 3.1 Kinetic Models Derived from the Full Experimental Data Set

All the kinetic models were trained with the full experimental data set (216 data points), and the RMSE values reported in Figure 2 are based on the 5-fold cross-validation results for the ML-based approaches and for the PLs. Only a selection of models is shown here for the sake of brevity. Among the various ML-based regression models, the 1-layer NN and GPR models show the lowest RMSEs. This is due to the structure of the models being better suited to describe intrinsically exponential trends. Decision tree models proved to be inadequate for describing the kinetic behavior, because of the binary nature of the method. Additionally, the 1-layer NN and the GPR performed better than the PL, as the latter in the reversible form tended to underestimate the reaction yield at temperatures above 280 °C. As for the irreversible PL model, it was intrinsically unsuitable for the description of the data set, as several points lay outside of the fully differential region (due to too high conversion at high temperature or low GHSV). The reparametrized LHHW kinetic model performs better than the PL, but shows a higher RMSE than the best ML-based methods, mainly because of the adaptation of one single parameter (*k* kinetic) to the new data set. However, one can observe that this approach yields good results despite its simplicity.

**Figure 2:**
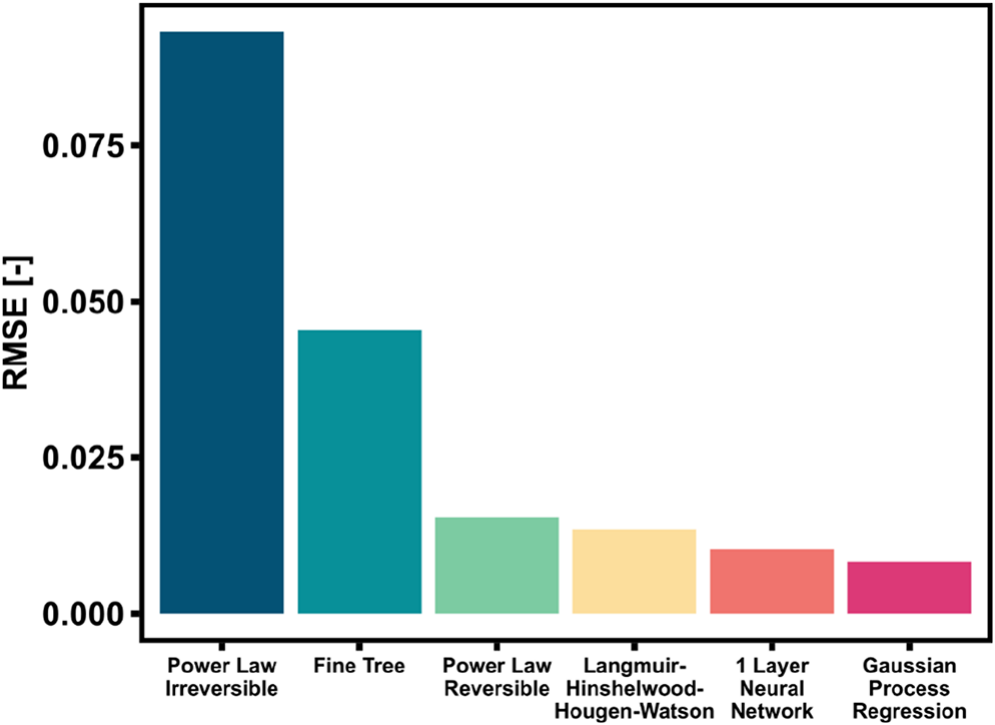
RMSE of different modeling approaches applied to the full experimental data set.

The different trends of the models were visualized by using parity plots. Figure 3 shows the comparison of the model predictions against the full experimental data set for PL reversible (a), NN (b), GPR (c) and LHHW (d). The dispersion of data for the PL model is evident, while the points for NN and GPR lie well on the perfect fit line. The LHHW model outperforms the PL in the entire interval considered, but the accuracy of the prediction is lower than the two ML-methods over the entire range of yield values considered. This is particularly surprising, because the PL model follows a well-established semiempirical correlation and the LHHW is based on a generally accepted mechanism for CO_2_ methanation, while ANN and GPR were derived without input of any physical information. In this sense, the amount of information required to derive the ANN and GPR models was significantly lower than the PL. Hence, the ML-based approach succeeded in finding mathematical correlations that describe well the experimental data points and satisfactorily pass the validation tests. The results should be anyway taken with care, as these results do not bring any evidence that the models could be used for extrapolation.

**Figure 3:**
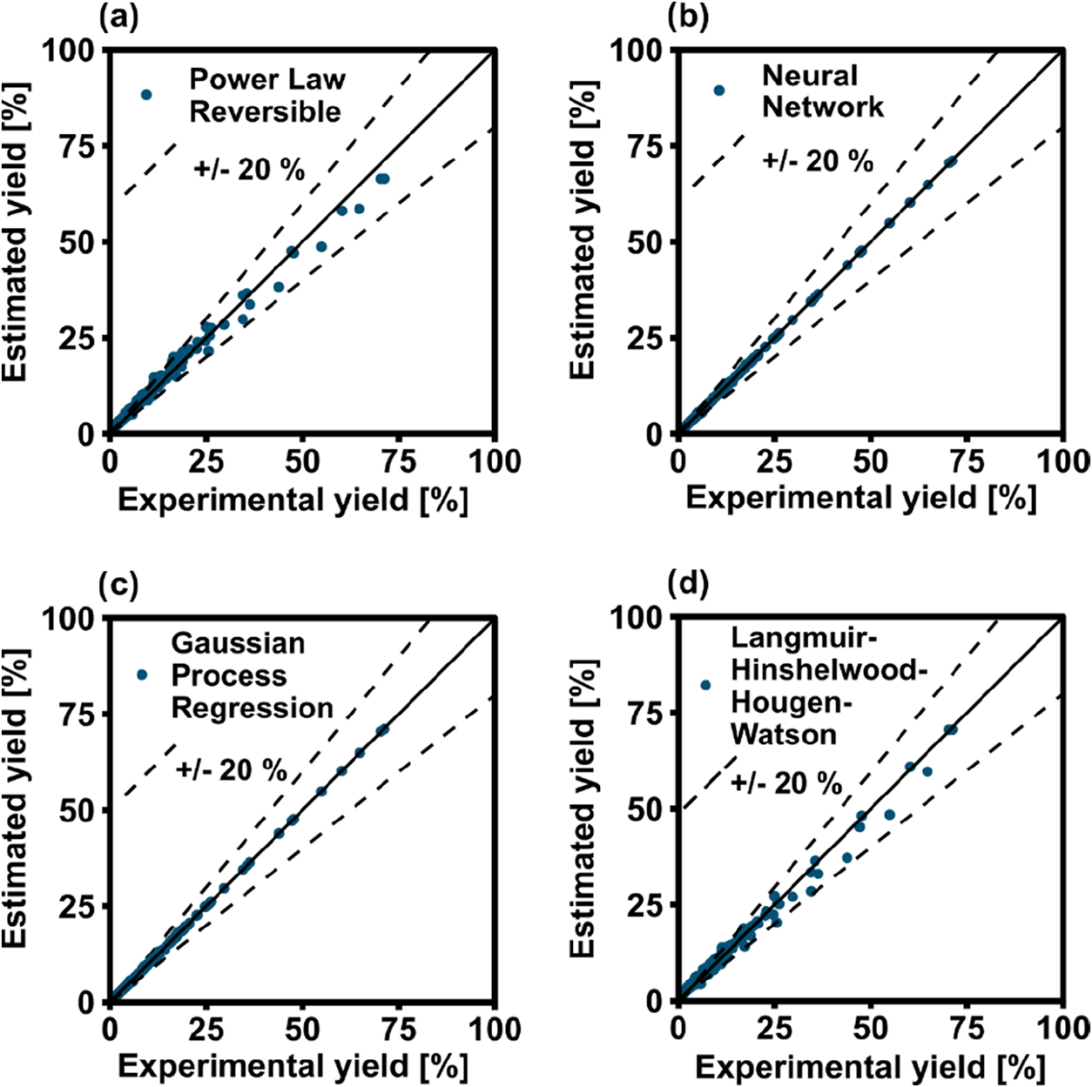
Parity plots of the (a) PL, (b) 1-layer NN, (c) GPR, (d) LHHW models applied to the full experimental data set.

### 3.2 Effects of the Parameter Choice on the Regression Performance

To assess the suitability of the models to predict the methane yield starting from different experimental data sets, the regressions were repeated using reduced data sets, in which relevant classes were removed. The following subsections report the results of two relevant examples.

#### 3.2.1 Limited Variation of H_2_/CO_2_ Ratio

The derivation of the PL model requires the knowledge of the conversion pattern over a large set of H_2_/CO_2_ ratios. Therefore, the reaction orders over the two reactants need to be derived. In principle, the ML-based models do not require this information, as they do not necessarily imply this correlation. For this reason, models were trained again with only the data points obtained close to stoichiometry (i.e., only the range of H_2_/CO_2_ between 3.75 and 4.25 was used). The predictive capacity of the obtained models was evaluated on said reduced data set in terms of RMSE (Figure 4) and parity plots (Figure 5). In this case, all the models performed slightly worse than the full data set, as all RMSEs are higher. The GPR is still the best regression method, while the ANN shows in this case a slightly higher RMSE compared to the PL. Looking at Figure 5, one can observe that the data points are more dispersed than in Figure 3, hinting that the lack of these data points worsens the quality of the kinetic model independently from the correlations used. We can hence conclude that the quality of the experimental data is essential for all the models investigated and that the PL is not particularly more affected by the lack of large H_2_/CO_2_ variations in the data set than the ML-based methods. Nevertheless, a ML-based method like the GPR has a better predictive performance than the PL.

**Figure 4:**
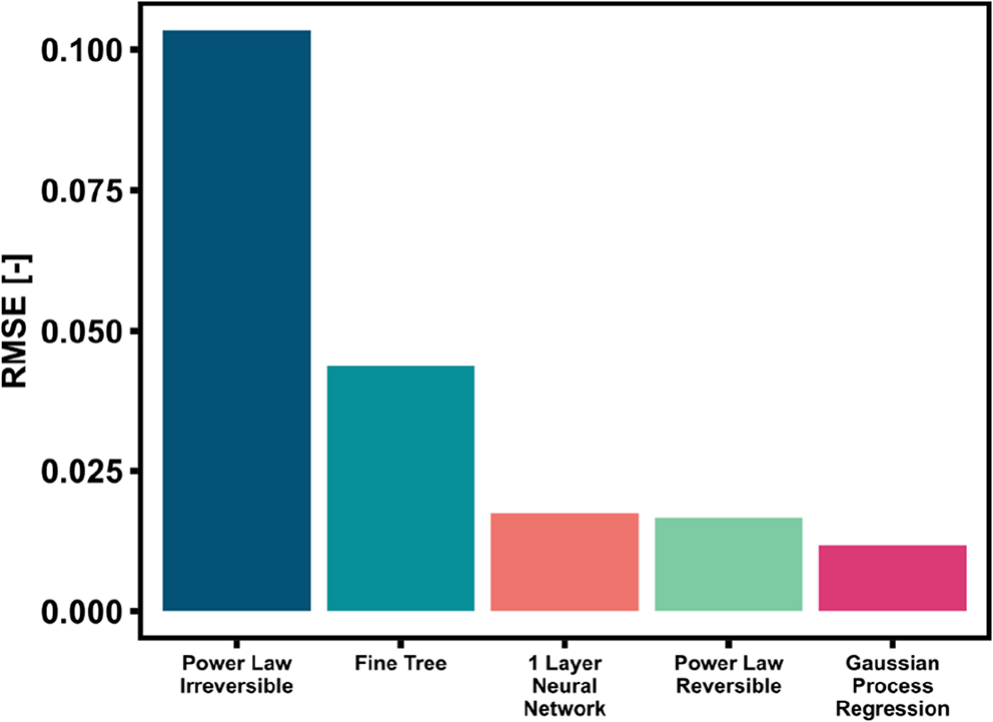
RMSE of the different models applied to a reduced data set obtained from the full data set without variation in stoichiometry.

**Figure 5:**
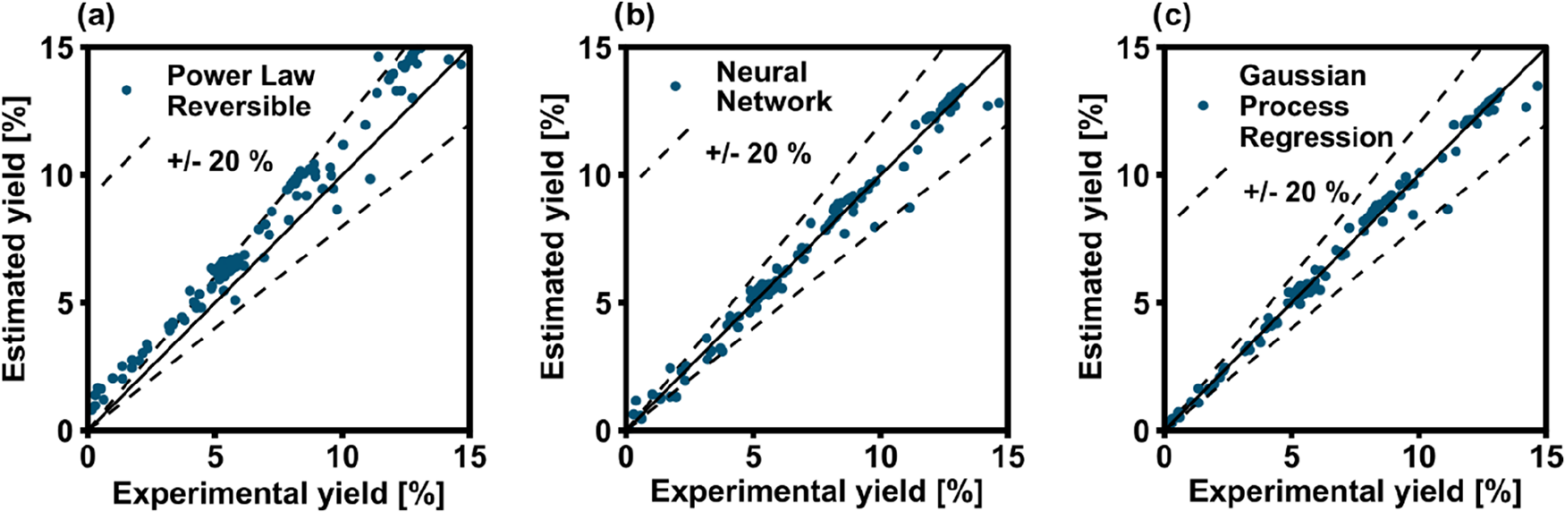
Parity plots of the PL trained with the (a) full stoichiometric data, as well as the (b) 1-layer NN and (c) GPR trained and applied to data sets lacking variations in stoichiometric ratio.

#### 3.2.2 Low CO_2_ Conversion Range

In the previous section, it was observed that the prediction capacity of the PL model worsens at higher conversions, leading to an overall higher RMSE than the ML-based models. For this reason, the performance of the regression models was evaluated when the complete data set was reduced by eliminating all the data points showing a conversion higher than 15% (fully differential data set). The rationale behind this choice was to simulate a region in which the PL model should perform well, while not giving the ML-based models any particular advantage. The results in terms of RMSE are shown in Figure 6 and as parity plots in Figure 7. Primarily, one can notice that the prediction is significantly better than in the previous cases, as the absolute RMSE values are significantly lower. Interestingly, the ML-based models still perform significantly better than the PL. This means that the correlations found by the ML-based approach are particularly well suited in describing the intrinsic kinetics of the methanation catalyst, resulting in a smaller prediction error than the standard PL-based model. This shows that ML-based models have a significant potential for kinetic modeling of highly selective catalysts like the material used in this study. It is likely that the PL could be improved via refinement of its parameters, but this would require additional analysis or experimental work.

**Figure 6:**
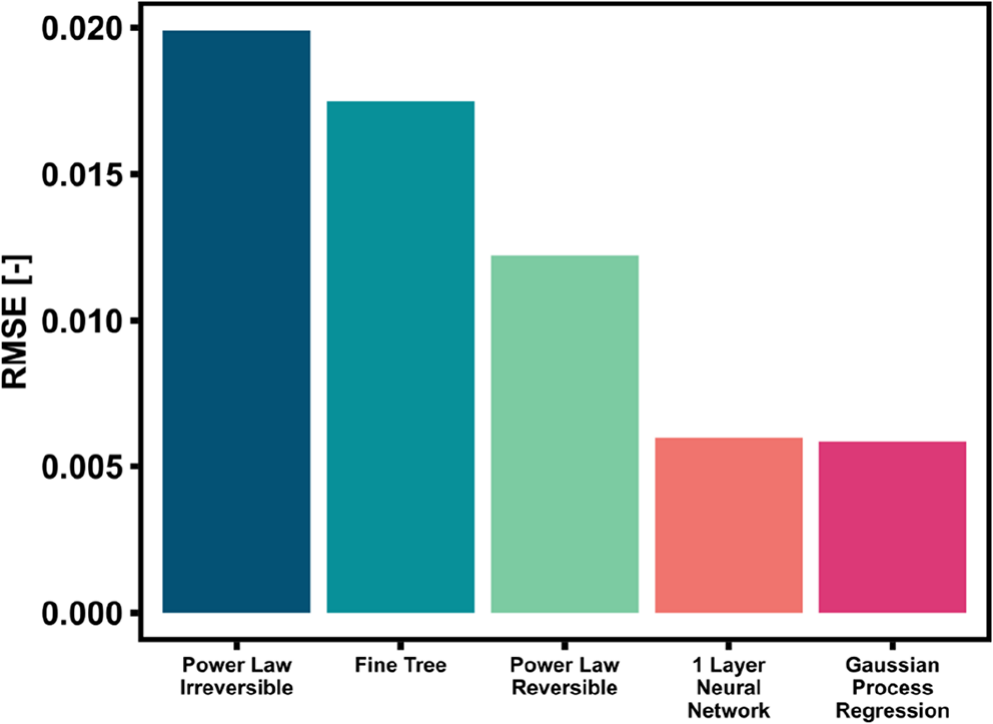
RMSE of models trained with data set 03 (<15% yield), applied to set 03 and 01 to evaluate the possibility to extrapolate the models.

**Figure 7:**
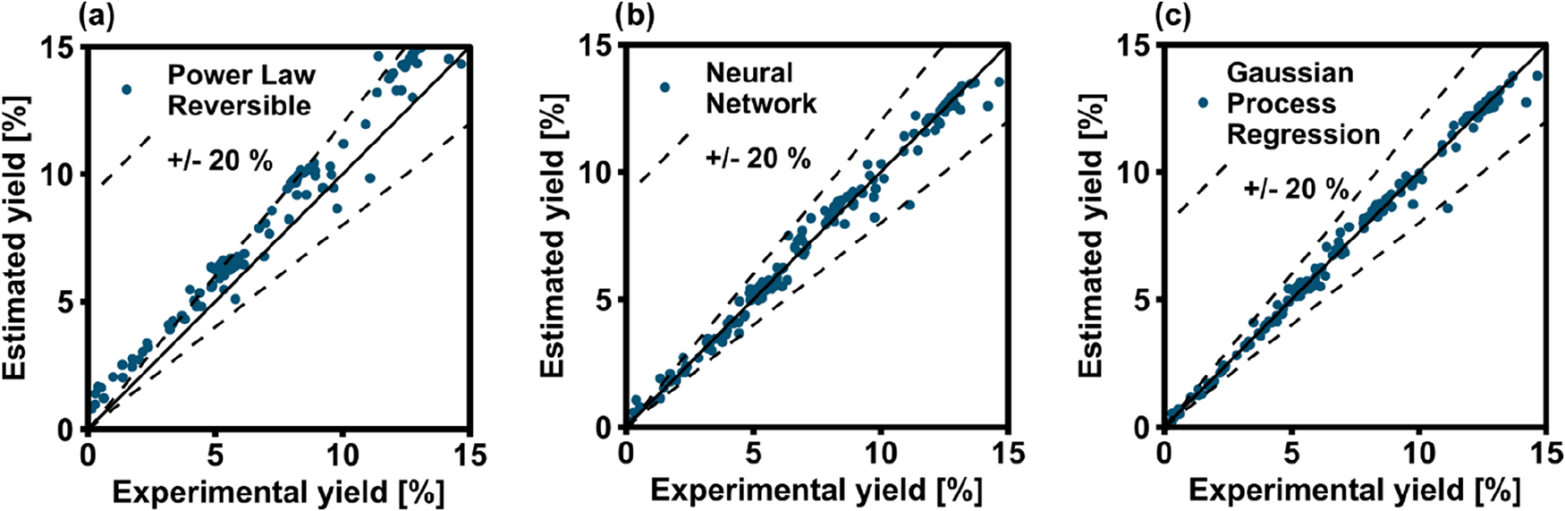
Parity plots of the (a) PL, as well as the (b) 1-layer NN and (c) GPR trained and applied to data set 03 (<15% yield).

#### 3.2.3 Summary of RMSE

A summary of RMSE obtained from the different models trained and applied to their respective data sets are given in Table 3, below, showing that the ML-based approaches tend to perform better than the PL, most notably the GPR model.

**Table 3:** RMSE of the PL, ANN, and GPR Approaches

data set	PL	1-layer NN	GPR
full set (01)	0.0154	0.0103	0.00831
near stoichiometry (02)	0.0166	0.0174	0.0118
<15% yield (03)	0.0113	0.00629	0.00557

### 3.3 Reactor Modeling

Until this point, the study focused on the capability of the kinetic models to describe the experimental data in terms of methane yield. However, one of the main applications of a kinetic model is the description of reaction kinetics in a reactor model, which is the scope of the following section. A first comparison of the kinetic modeling approaches with referencing the experimental data of one temperature ramp is shown in Figure 8. The parameters of the PL and GPR kinetic models were derived by regression over the complete kinetic data set, as detailed above. As for the LHHW model, the three parameters out of four were taken from literature and not derived from the kinetic data set. The LHHW model from literature fits very closely to the experimental data point, following the kinetic region in its entirety. The selected GPR model also describes the experimental data points well, with a similar trend as the LHHW approach. The PL also describes relatively well the experimental data points, but the average prediction error is higher, especially at higher temperatures. This first comparison suggests that all the selected kinetic model types are suitable in describing the performance of the industrial catalyst in the kinetics limited parametric region.

**Figure 8:**
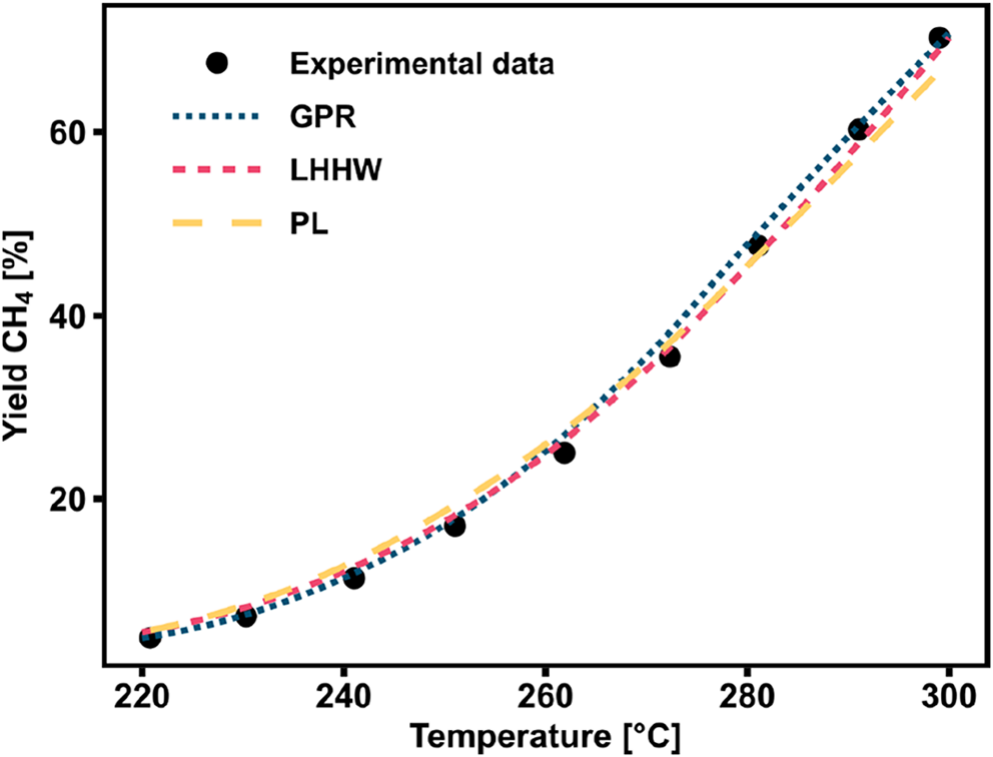
Methane yield as a function of temperature for the experimental data, LHHW from Koschany et al.^16^ and an ML-based regression and PL (experimental conditions: *m*_cat_ = 0.720 g, *P* = 3 bar, GHSV = 110,000 h^–1^).

#### 3.3.1 Black Box Model

For most practical applications of kinetic models, including reactor modeling and simulation, the description of kinetics in areas where the reaction rate goes down to zero due thermodynamic equilibrium is essential. In the “black box model”, the conversion at the thermodynamic equilibrium was added as supplementary experimental data points to the training and test data set of the ML-driven surrogate models. Additionally, the same points were used in the testing data set of the PL. This data set uses thermodynamic equilibrium data calculated via minimization of the Gibbs free energy coupled with low GHSV values (<1000 h^–1^). In this way, the ML-based models are trained to learn the conversion at the thermodynamic equilibrium. The model hence predicts the conversion over a large range of temperature and GHSV, following a black box approach (not predicting the actual conversion profile in the reactor).

The results of the predictions are displayed in Figure 9 as parity plots. The GPR model performs best in terms of prediction efficacy. The predicted data points lie very close to the actual results over the entire parameter range evaluated. The ANN also shows a good performance in the prediction of the yield, with some signs of overfitting at high conversion. This issue could be mitigated by using an alternative training set at the thermodynamic equilibrium. The splitting of the full data set during training may have produced an uneven or insufficient representation of the thermodynamic equilibrium. While the PL is effective at predicting the conversion at the thermodynamic equilibrium, it is less accurate in the 50–75% yield range. These results suggest that the ML-based models derived in this study are effective in describing the CO_2_ conversion obtained over the selected catalyst as a function of GHSV and temperature. This type of information is sufficient for a simple reactor design and hence can already support the scale up of a process based on this catalyst. The ML-based models therefore show potential in being employed for black-box reactor design, despite the low experimental effort required in obtaining them.

**Figure 9:**
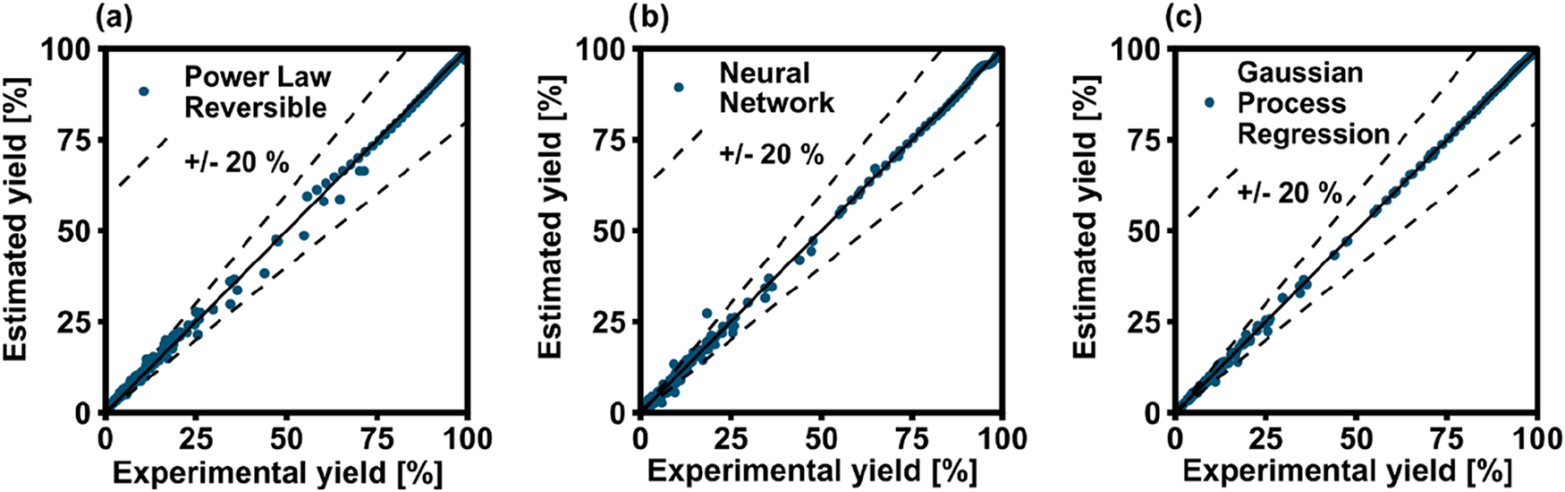
Parity plots of the (a) PL, (b) 1-layer NN and (c) GPR trained and applied to data set 04 (full data set, supplemented with thermodynamic equilibrium data points).

#### 3.3.2 Isothermal Reactor Model

Once the performance of the kinetic models in black box modeling approach had been determined, their performance in the simulation of an isothermal reactor was assessed and compared. This causes a great increase in the complexity of the prediction, as in this case the models need to describe the reaction rate over the entire set of parameters that change over the axial coordinate of a reactor. Figure 10 shows the results in terms of predicted methane yield at the reactor outlet. Panel (a) shows the simulations performed with the ML-based regression (GPR), while panel (b) reports the results of the modeling performed with the standard LHHW-based approach. The dotted points are representative of the experimental data points in the central region of the kinetic regression, which should be predicted satisfactorily by the kinetic models.

**Figure 10:**
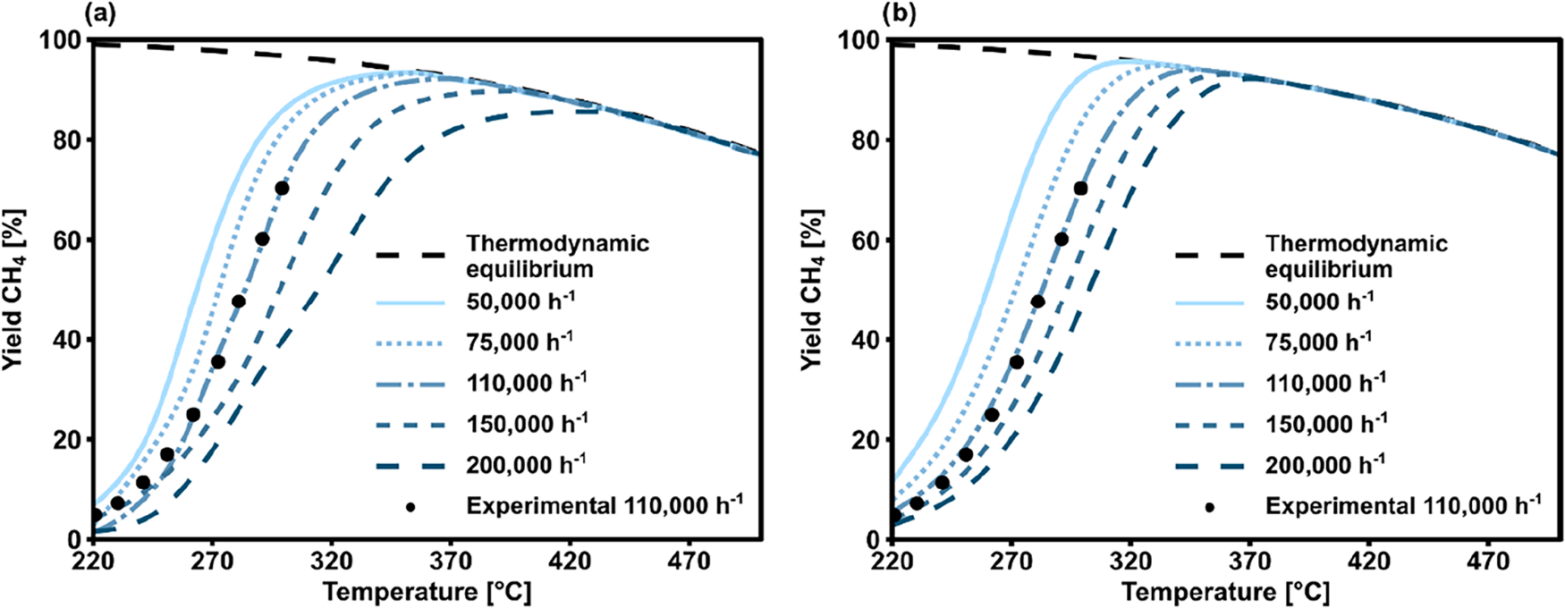
Methane yield as a function of temperature in the isothermal reactor model using kinetics derived with (a) GPR and (b) LHHW model, alongside experimental points (GHSV = 110,000 h^–1^).

The results of the modeling follow the expected trends, with the whole temperature range covered by three distinct regions: the purely kinetic region, with the yield increasing exponentially with the temperature, an intermediate region, where the proximity with the thermodynamic equilibrium reduces the increasing trend of yield with temperature and a thermodynamically limited region, where the methane yield decreases with temperature. Both models describe the central kinetic region of the graphs well, including the experimental data points. The effect of the gas hourly space velocity in this region is described in a similar way by the two models. The LHHW-based approach follows the thermodynamic equilibrium curve well, which is expected from this established modeling method. Directly applying the black-box approach of the ML-based regression derived before did not give satisfactory results. This was due to an overestimation of the conversion at the thermodynamic equilibrium points, originating from a residual reaction rate predicted by the models after reaching the equilibrium curve. For this reason, it was necessary to perform additional training of the ML-based model, using both the thermodynamic equilibrium curve in terms of equilibrium conversion and nil reaction rate. After applying this procedure, the description of the thermodynamic equilibrium range also became satisfactory for the ML-based kinetic model. In this way, the GPR model derived here appears as a good option for the description of the isothermal reactor.

To better understand the suitability of the model for extrapolation, the two models were compared in detail by means of a parity plot. The parity plot is shown in Figure 11. The color scale reflects the space velocities employed in Figure 10. For most of the simulation curves, clear trends can be found. The kinetic region is described in a similar way by the two models, with minimal discrepancies (e.g., a slightly lower yield is predicted by GPR for the region below 230 °C). This is an expected result as this is the original area of training of the models with the experimental data. Hence, both models are supposed to describe this region well, as already demonstrated in the previous sections. The distance between the two models tends to increase when the model gets far from the central regression zone, showing that the extrapolation of the ML-based kinetic model could become critical. However, in the GHSV range investigated, the discrepancies between the LHHW- and the ML-based kinetic models are limited.

**Figure 11:**
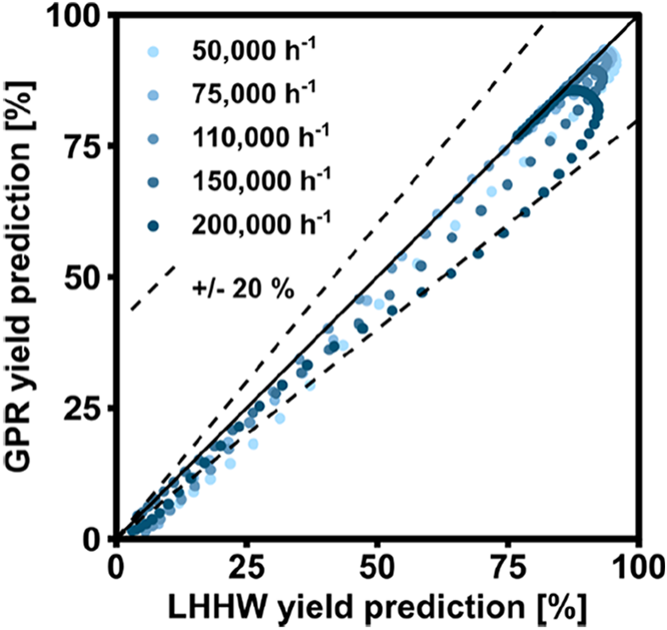
Parity plot of the values predicted using the ML-based GPR approach vs those obtained from the standard LHHW model.

The larger discrepancies between the two models are found in the intermediate region, where the influence of the thermodynamic equilibrium is described is a slightly different way. The LHHW-based model seems let the reaction rate grow exponentially, before bending to the thermodynamic equilibrium very close to the equilibrium curve. The ML-based model, however, tends to bend the curve earlier, thus reaching the thermodynamic equilibrium at a higher temperature. This phenomenon is most pronounced for the case at the highest GHSV, where the underestimation of conversion of the ML-based model compared to the LHHW approach reaches the set 20% limit. Interestingly, this phenomenon is in line with what was observed for catalysts subject to significant diffusional limitations, where the efficiency factor in this region tends to be low, as observed both experimentally^35^ and by modeling^36^ in literature.

In conclusion, the GPR derived in this section is well suited to predict the methane yield in a wide range of flow ranges. When compared to a LHHW-based kinetic model implemented in the same reactor model, both predictions are very close in the central region of the regression, with some discrepancies found when moving away from this region. However, the difference in predicted methane yield is limited in the 50,000–200,000 h^–1^ GHSV range, which appears sufficiently accurate for practical applications. Additionally, it should be noted that the data used for the training of the ML-based model were obtained in a simple setup where the conversion could be measured only at the reactor outlet. For this reason, the range in which true differential data could be obtained was limited. It would therefore be possible to improve the predictive capabilities of these models with the use of spatially resolved data points, from which the reaction rate could be derived more precisely.

#### 3.3.3 Nonisothermal Reactor Model

The prediction of temperature and concentration profiles in an isothermal reactor is a good test for the quality of kinetic models. However, in the real world, nonisothermal reactors prevail, for which the kinetic model has to be used. To understand whether the ML-based kinetic model derived are suitable for the prediction of the temperature and concentration profiles of a real reactor, the GPR model was implemented in an existing reactor model, aimed at describing the performance of an industrial scale reactor, whose temperature profile is available in literature.^28^ The reactor is a plate-type heat exchanger, filled with the same catalyst used for the kinetic model elucidated in this study. The model used is explained in the Section 2.

The results of the simulations are shown in Figure 12, highlighting the differences between the GPR kinetic model and a standard LHHW kinetic model from literature. The GPR kinetic model appears to provide better results than the LHHW kinetic model as it provides a better fit to the experimental temperature profile. The discrepancies in the model performance can be explained as follows. As highlighted in the previous section, the GPR model was derived using only low conversion data. To adequately describe the decline in reaction rate when approaching the equilibrium conditions, the data set was supplemented with thermodynamic equilibrium data. For the intermediate conversion range between the kinetic region and thermodynamic equilibrium (i.e., ∼400–500 °C, ∼75–90% CO_2_ conversion), the GPR model regression does not rely on experimental data. Hence, this model tends to underestimate the conversion compared to the LHHW model in this range. This is directly reflected in the reactor model, where the reaction rate decreases prior to the hotspot, resulting in the blue temperature profile being shifted to a later axial coordinate. This can be visualized in terms of CO_2_ conversion, as reported in Figure 13, where the predicted conversion in the first 5% of the reactor is higher for the LHHW than the GPR kinetic model. As for the slightly higher hotspot temperature of the GPR model, this instead originated from the slightly different thermodynamic equilibrium positioning.

**Figure 12:**
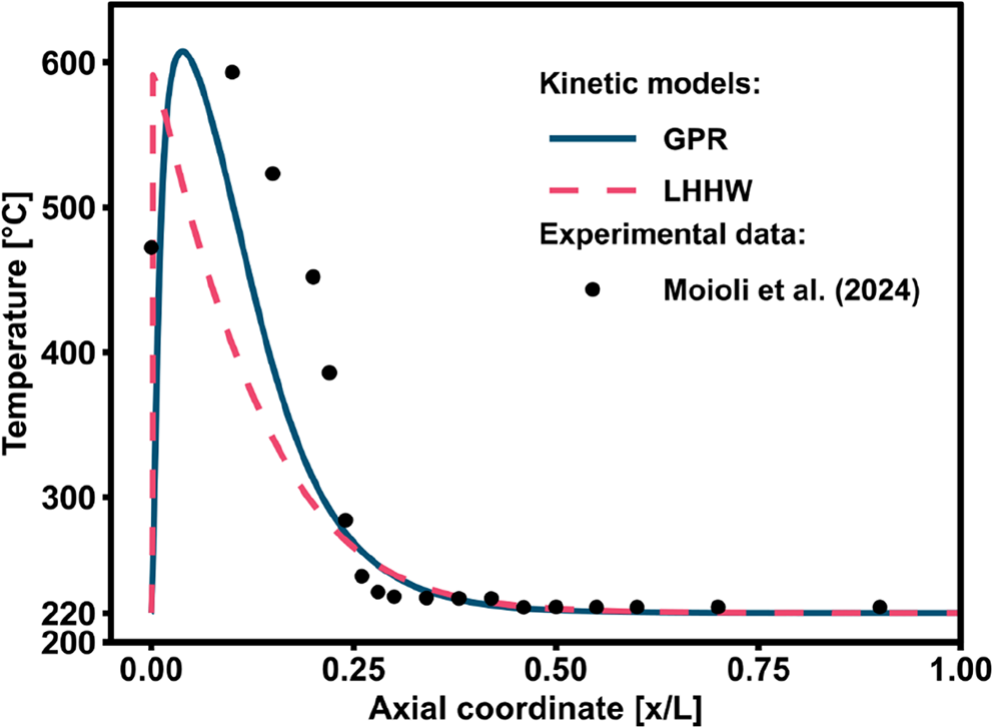
Comparison of the temperature profiles obtained from the adiabatic reactor model using the LHHW or GPR models and the experimental data.^28^

**Figure 13:**
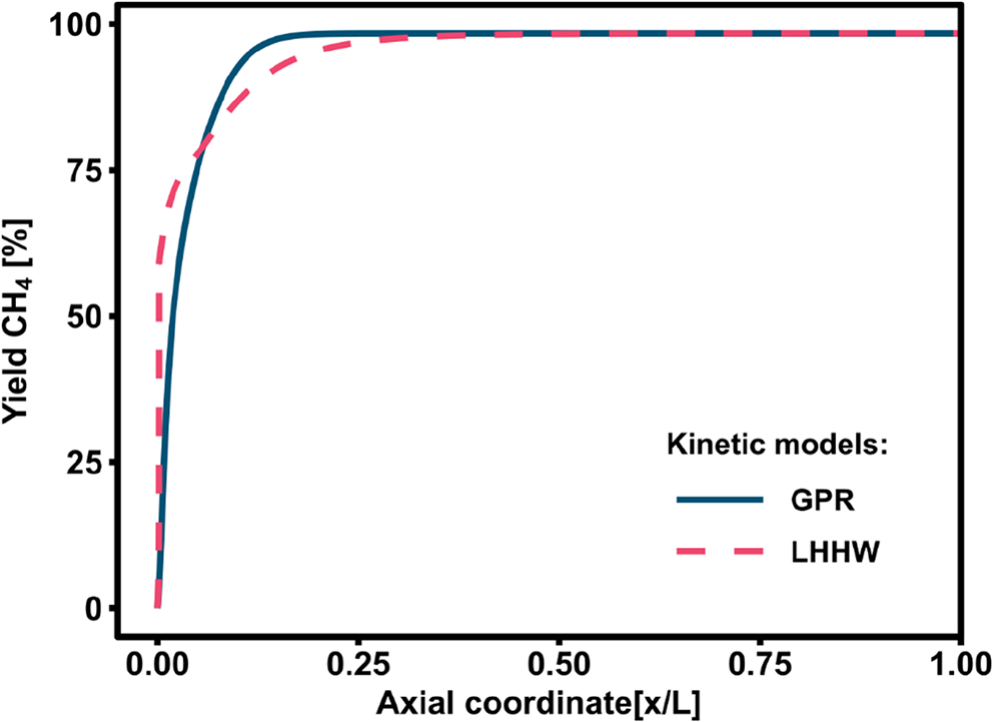
Methane yield vs axial coordinate in an adiabatic reactor for the LHHW or GPR models.

Downstream of the hotspot, the GPR predicts a higher residual reaction rate than the LHHW kinetics, hence requiring a larger reactor volume, and consequently longer axial coordinate space, to decrease the temperature. Interestingly, the GPR model better approximates the experimental data, most probably because the real reaction rate is slowed down by the influence of intraparticle diffusional limitations and by axial heat dispersion in the first part of the reactor. These phenomena should be better taken into account by the reactor model, which could improve the overall predictive performance of the LHHW kinetic rate equations. In the later part of the reactor (*x*/*L* > 0.25), the reaction rate is slow and the performance is determined by the thermodynamic equilibrium term of the reactor model, leading to identical temperature profiles for both approaches.

Overall, this simple case study demonstrates that ML-based regressions can describe reaction kinetics across a wide range of parameters at least as well as classical approaches. This study hints that certain ML-based regressions have the potential to correctly interpret nonideal behaviors, based on the data used for regression. However, it is evident that the potential of these methods is strongly linked to the quality of the data supplied and that the capability to predict the kinetic behavior of complex systems may be limited. For this reason, the methods proposed in this study should be carefully tested in more complex model reactions in the future, to ensure their suitability for general kinetic modeling purposes.

## 4 Conclusions

In this study, an experimental data set of methane yields over a commercial CO_2_ methanation catalyst was obtained. Several kinetic models were derived from this data, using standard approaches (PL and LHHW) alongside ML-based approaches. The high activity and excellent selectivity of the catalyst toward methane allowed the system to be described using a single rate equation. This was ideal as it enables an improved understanding of the effectiveness of the models. Additionally, the performance of a technical reactor could be adequately described by the kinetic models. This was done by comparing the prediction of a reactor model with experimental data obtained from a full-scale reactor.

All the standard kinetic models tested described the entire kinetic data set satisfactorily. The kinetic data can be explained using a reasonable number of parameters and the calculated activation energy as well as the reaction orders are in line with literature data. Among the ML-based approaches, 1-layer ANNs and GPRs suitably describe the entire kinetic data set with a low average prediction error. The selected modeling approaches were further evaluated by modifying the structure of the data set, to understand whether certain types of parameter variations are essential to obtain a reliable kinetic model with both model types. The reduction of the range of H_2_/CO_2_ variations is detrimental for the prediction capacity of the PL, as expected. Interestingly, the ML-based models are also penalized by the absence of such data, showing that the information carried by this parameter variation is essential independently of the regression strategy employed. The reduction of the data set to a conversion range below 15% (i.e., to essentially differential conditions) improved the prediction capacity of the models, showing that all the models used are suitable for the description of differential data. This is an interesting result, as ML-based models are good in predicting the conversion in differential conditions even though they are not supplemented with data concerning the chemistry of the reaction.

Concerning the reactor modeling, the ML-based models show good performance in describing the reactor in a black-box approach (i.e., predicting conversion as a function of temperature, pressure, composition and space velocity), even in conditions characterized by thermodynamic equilibrium. To predict the equilibrium conversion, it is sufficient to train the models with a kinetic data set complemented by thermodynamic data (i.e., by including the equilibrium conversion as the result of the reaction at low GHSV). The PL approach is less effective in the prediction with a black box approach, as determined by a higher RMSE.

When describing an isothermal reactor, the GPR model must be complemented by further training data, forcing the reaction rate to 0 when the conversion reaches the equilibrium conditions. In this way, the kinetic model implemented in an isothermal reactor model gives acceptable results in a relatively large interval around the training points. When compared to a state-of-the-art LHHW model for a GHSV comprised between 50,000–200,000 h^–1^, the difference in prediction is limited. This can be regarded as a good result for the simple ML-based kinetic model employed, as the LHHW approach requires much more in-depth knowledge of the catalytic system.

The prediction performance of the GPR model when implemented in a nonisothermal reactor model is surprisingly good. The GPR model outperforms the LHHW approach in the prediction, most likely due to the data-driven approach extrapolating some nonideal features (e.g., diffusional limitations). These already being present at the lab scale but becoming evident at the reactor scale. The results reported here open the way for additional investigation that should aim at a full understanding of this phenomenon. Future works will hence be aimed at the investigation of the effect of ML-based kinetic models on the modeling of technical reactors.

## Supplementary Material



## Notes

The full experimental data set, explanation on the procedure to perform to obtain the ML-based models, diagram of the reactor setup used for the kinetic measurements and experimental space explored during the kinetic study (PDF)

## Table of Abbreviations

Abbreviation: full description
AI: artificial intelligence
ANN: artificial neural network
bw: boiling water
GHSV: gas hourly space velocity
GPR: Gaussian process regression
LHHW: Langmuir–Hinshelwood-Hougen-Watson
ML: machine learning
MS: mass spectrometer
NDIR: nondispersive infrared sensor
NN: neural network
pb: packed bed
PL: power law
ReLU: rectified linear unit
RMSE: root mean squared error

## Table of Symbols

Symbol: variable units
*A*: reactor cross-section m^2^
cp,mix: mass-specific heat of gas mixture J·kg^–1^·K^–1^
*Ea*_*j*_: activation energy of reaction *j* J·mol^–1^
*f*_Corr_: correction factor for kinetic models -
*F*_*i*_: component *i* molar flow rate kmol·s^–1^
*h*: convective heat transfer coefficient W·m^–2^·K^–1^
*k*_*j*_: kinetic constant of reaction *j* (β depending on mole balance of reaction) kmol·s^–1^·kg_c_^–1^ Pa^β^
*K*_*j*_^eq^: equilibrium constant of reaction *j* (β depending on mole balance of reaction) Pa^β^
*l*_g_: heat conductibility of the gas W·m^–1^·K
*ṁ*: total mass flow rate kg·s^–1^
*Nu*: Nusselt number -
*p*_*i*_: partial pressure of component *I* Pa or bar
*Pr*: Prandtl number -
*Re*: Reynolds number -
*r*_*j*_: reaction rate of reaction *j* kmol·kg^–1^·s^–1^
*T*: temperature *K*
*T*_e_: temperature of external coolant *K*
*U*: global heat transfer coefficient W·m^–2^·K^–1^
*u*_G_^0^: gas superficial velocity m·s^–1^
*W*: width of fixed-bed reactor channels *m*
*z*: axial coordinate in the reactor m
*a*, β: catalyst activity -
Δ*h*_j_^R^: reaction enthalpy of reaction *j* J·mol^–1^
ε: overall void fraction -
ν_*ij*_: stoichiometric coefficient of species *i* in reaction *j* -
ρ_Cat_: catalyst density kg·m^–3^
