# Supplementary material for: Machine Learning-Based Kinetic Modeling of the CO2 Methanation Reaction over an Industrial Catalyst
Source: Ind Eng Chem Res. 2025 Oct 4;64(41):19864–75. doi: 10.1021/acs.iecr.5c02947 (PMC12532210; doi:10.1021/acs.iecr.5c02947)
Supplement: Supplementary file 1 [file ie5c02947_si_001.pdf]

Supplementary material for:

Machine learning-based kinetic modelling of the CO_2 methanation reaction over an industrial catalyst

Hugo Pétremand[1,2], Julia Witte[3], Oliver Kröcher[1,2], Emanuele Moioli[1,4]*

[1]PSI Center for Energy and Environmental Sciences, Paul Scherrer Institute, 5232 Villigen PSI, Switzerland.

[2]Institute of Chemical Sciences and Engineering, Ecole Polytechnique de Lausanne, 1015 Lausanne, Switzerland.

[3]Kanadevia Inova AG, Hardturmstrasse 127, 8005 Zürich, Switzerland.

[4]Dipartimento di Chimica, Materiali e Ingegneria Chimica 'Giulio Natta', Politecnico di Milano, 20133 Milano, Italy.

*Corresponding author: emanuele.moioli@polimi.it

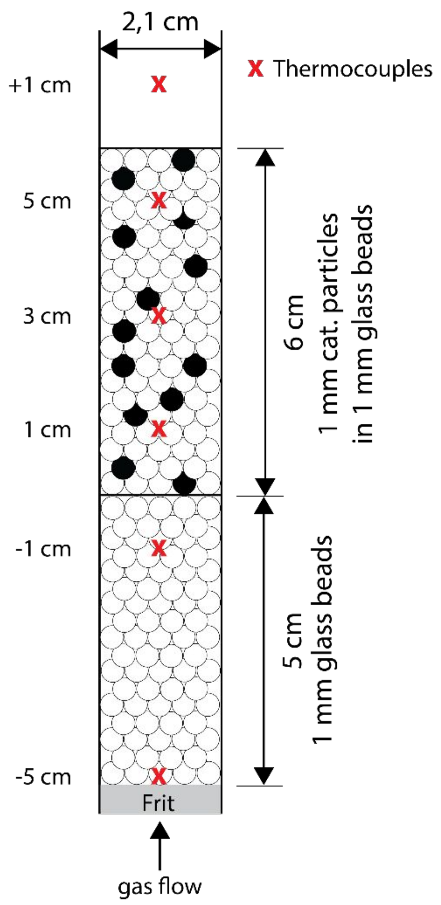

Figure S1. Diagram of the reactor setup used for the kinetic measurements.

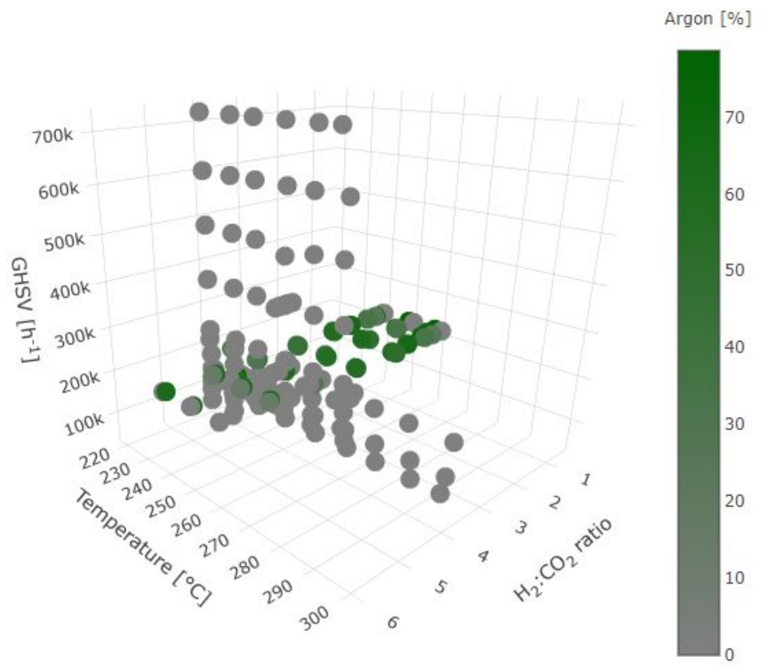

Figure S2. Experimental space explored during the kinetic study.

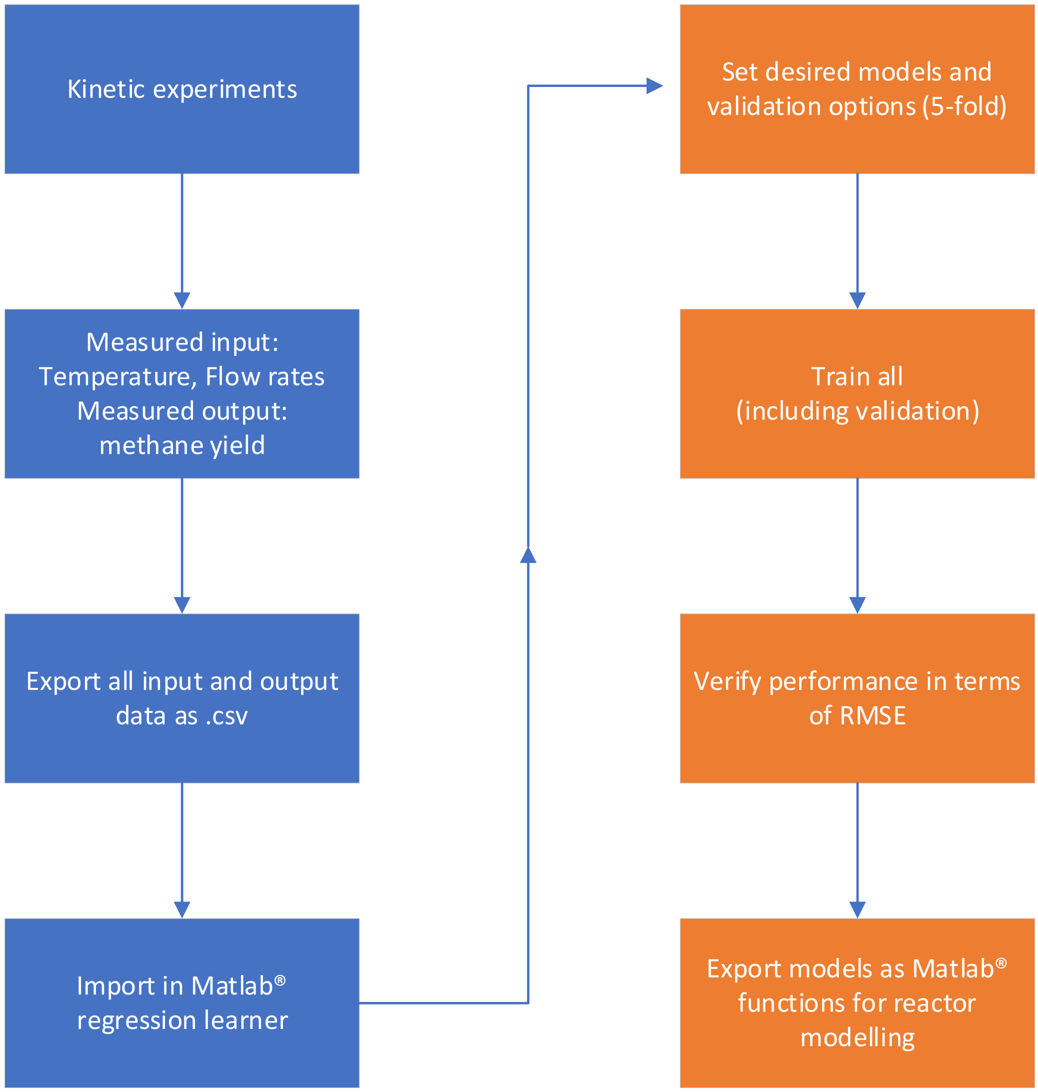

Figure S3. The workflow applied in this work.

Table S1. Experiments performed for the kinetic study.

Test #	Temperature [°C]	CO_2 [%]	H_2 [%]	Ar [%]	GHSV [h-1]	Flowrate [NmL/min]	CH_4 yield [%]
1	240	20.0	80.0	0.0	1.44E+05	599	11.09
2	251	20.0	80.0	0.0	1.44E+05	599	17.27
3	261	19.9	80.1	0.0	1.44E+05	599	25.56
4	272	20.0	80.0	0.0	1.44E+05	599	34.50
5	280	20.0	80.0	0.0	1.44E+05	599	43.86
6	290	20.0	80.0	0.0	1.44E+05	599	54.88
7	300	20.0	80.0	0.0	1.44E+05	599	64.75
8	231	20.0	80.0	0.0	7.20E+05	3000	0.29
9	230	20.0	80.0	0.0	6.00E+05	2500	0.51
10	231	20.0	80.0	0.0	4.80E+05	2000	0.98
11	231	20.0	80.0	0.0	3.60E+05	1499	1.75
12	231	20.0	80.0	0.0	2.40E+05	1000	3.34
13	230	20.1	79.9	0.0	1.78E+05	742	4.38
14	230	20.0	80.0	0.0	1.20E+05	500	7.12
15	230	20.0	80.0	0.0	9.00E+04	375	9.57
16	230	20.0	80.0	0.0	6.00E+04	250	14.66
17	230	19.9	80.1	0.0	4.78E+04	199	18.44
18	230	20.0	80.0	0.0	1.44E+05	599	5.12
19	250	20.0	80.0	0.0	7.20E+05	3000	2.35
20	251	20.0	80.0	0.0	6.00E+05	2500	3.21
21	250	20.1	79.9	0.0	4.62E+05	1923	4.01
22	250	20.0	80.0	0.0	3.60E+05	1499	5.62
23	251	20.0	80.0	0.0	2.40E+05	999	8.58
24	250	20.0	80.0	0.0	1.69E+05	704	11.41
25	250	20.0	80.0	0.0	1.44E+05	599	14.18
26	250	20.0	80.0	0.0	1.20E+05	499	16.59
27	251	20.0	80.0	0.0	2.40E+05	1000	9.65
28	271	20.0	80.0	0.0	1.20E+05	500	36.36
29	270	20.0	80.0	0.0	1.44E+05	599	29.68
30	271	20.0	80.0	0.0	1.80E+05	749	24.68
31	270	20.0	80.0	0.0	2.40E+05	1000	18.76
32	270	20.0	80.0	0.0	3.60E+05	1499	12.75
33	269	19.9	80.1	0.0	4.85E+05	2022	9.24
34	270	20.0	80.0	0.0	6.00E+05	2500	7.89
35	262	20.0	80.0	0.0	7.20E+05	3000	5.79
36	230	20.0	80.0	0.0	1.44E+05	599	5.72
37	231	20.0	80.0	0.0	1.44E+05	599	5.91
38	231	19.6	80.4	0.0	1.44E+05	600	5.86
39	230	19.3	80.7	0.0	1.44E+05	600	5.87
40	230	19.6	80.4	0.0	1.44E+05	600	5.87
41	230	19.9	80.1	0.0	1.44E+05	599	5.64
42	230	20.4	79.6	0.0	1.44E+05	601	5.36
43	230	20.9	79.1	0.0	1.44E+05	600	5.32
44	230	20.4	79.6	0.0	1.44E+05	600	5.34
45	230	20.0	80.0	0.0	1.44E+05	599	5.62
46	250	20.0	80.0	0.0	3.60E+05	1499	5.56
47	250	19.6	80.4	0.0	3.60E+05	1500	5.54
48	250	19.2	80.8	0.0	3.60E+05	1499	5.57
49	250	19.6	80.4	0.0	3.60E+05	1500	5.39

Test #	Temperature [°C]	CO_2 [%]	H_2 [%]	Ar [%]	GHSV [h-1]	Flowrate [NmL/min]	CH_4 yield [%]
50	250	20.0	80.0	0.0	3.60E+05	1500	5.22
51	250	20.4	79.6	0.0	3.60E+05	1500	5.18
52	250	20.8	79.2	0.0	3.60E+05	1499	5.17
53	250	20.4	79.6	0.0	3.60E+05	1500	5.36
54	250	20.0	80.0	0.0	3.60E+05	1500	5.33
55	230	20.0	80.0	0.0	1.44E+05	600	5.83
56	230	20.0	80.0	0.0	1.44E+05	599	5.80
57	221	20.0	80.0	0.0	1.44E+05	599	3.34
58	221	20.0	80.0	0.0	1.20E+05	500	4.16
59	220	20.0	80.0	0.0	1.80E+05	749	2.32
60	220	20.0	80.0	0.0	2.40E+05	999	1.35
61	220	20.0	80.0	0.0	3.60E+05	1500	0.39
62	220	19.9	80.1	0.0	4.83E+05	2013	0.62
63	219	20.0	80.0	0.0	6.00E+05	2500	0.29
64	219	20.0	80.0	0.0	7.20E+05	3000	0.15
65	220	20.0	80.0	0.0	1.20E+05	500	4.21
66	220	20.0	80.0	0.0	9.00E+04	375	5.93
67	220	20.0	80.0	0.0	6.00E+04	250	9.22
68	239	20.0	80.0	0.0	1.20E+05	500	10.03
69	241	20.0	80.0	0.0	1.44E+05	599	8.94
70	241	20.0	80.0	0.0	1.80E+05	749	6.96
71	240	20.0	80.0	0.0	2.40E+05	1000	5.13
72	240	20.0	80.0	0.0	3.60E+05	1499	3.17
73	240	20.0	80.0	0.0	4.80E+05	2000	2.15
74	240	20.0	80.0	0.0	6.00E+05	2500	1.74
75	240	20.0	80.0	0.0	7.20E+05	3000	1.36
76	230	20.0	80.0	0.0	1.44E+05	599	5.50
77	230	20.0	80.0	0.0	1.44E+05	599	4.96
78	230	20.0	80.0	0.0	1.44E+05	599	5.70
79	260	20.0	80.0	0.0	1.20E+05	500	22.80
80	260	20.0	80.0	0.0	1.44E+05	599	19.62
81	260	20.0	80.0	0.0	1.80E+05	749	15.93
82	260	20.0	80.0	0.0	2.40E+05	999	12.31
83	260	20.0	80.0	0.0	3.60E+05	1499	8.20
84	260	20.0	80.0	0.0	4.80E+05	2001	6.15
85	260	20.0	80.0	0.0	6.00E+05	2500	4.85
86	261	20.0	80.0	0.0	7.20E+05	3000	4.28
87	230	20.0	80.0	0.0	1.44E+05	599	6.12
88	230	20.1	79.9	0.0	2.15E+05	897	3.80
89	230	15.9	63.6	20.5	2.17E+05	903	4.45
90	230	11.9	47.3	40.8	2.19E+05	911	5.34
91	230	8.0	32.0	60.0	2.16E+05	899	6.91
92	230	5.0	19.7	75.3	2.15E+05	897	9.79
93	230	20.0	80.0	0.0	1.44E+05	599	5.71
94	230	20.0	80.0	0.0	1.44E+05	599	5.71
95	231	20.0	80.0	0.0	2.15E+05	897	3.70
96	220	20.1	79.9	0.0	2.15E+05	897	2.00
97	240	20.0	80.0	0.0	2.15E+05	897	5.60
98	251	20.0	80.0	0.0	2.15E+05	897	8.93
99	260	20.0	80.0	0.0	2.15E+05	897	12.93
100	272	20.1	79.9	0.0	2.15E+05	897	20.65

Test #	Temperature [°C]	CO_2 [%]	H_2 [%]	Ar [%]	GHSV [h-1]	Flowrate [NmL/min]	CH_4 yield [%]
101	279	20.1	79.9	0.0	2.15E+05	897	26.20
102	301	20.0	80.0	0.0	2.15E+05	897	47.13
103	289	20.1	79.9	0.0	2.15E+05	897	34.44
104	281	19.9	80.1	0.0	1.08E+05	450	47.64
105	291	19.9	80.1	0.0	1.08E+05	450	60.28
106	299	19.9	80.1	0.0	1.08E+05	450	70.27
107	299	19.9	80.1	0.0	1.08E+05	450	71.32
108	231	19.9	80.1	0.0	1.44E+05	599	5.82
109	221	19.9	80.1	0.0	1.08E+05	450	4.88
110	230	19.9	80.1	0.0	1.08E+05	450	7.22
111	241	19.9	80.1	0.0	1.08E+05	450	11.35
112	251	19.9	80.1	0.0	1.08E+05	450	17.04
113	262	19.9	80.1	0.0	1.08E+05	450	24.99
114	272	19.9	80.1	0.0	1.09E+05	455	35.54
115	241	19.9	80.1	0.0	1.44E+05	599	8.78
116	241	19.6	80.4	0.0	1.44E+05	600	8.83
117	241	19.2	80.8	0.0	1.44E+05	600	8.89
118	241	19.6	80.4	0.0	1.44E+05	600	8.70
119	241	20.0	80.0	0.0	1.44E+05	599	8.48
120	241	20.5	79.5	0.0	1.44E+05	600	8.23
121	241	20.8	79.2	0.0	1.44E+05	600	8.16
122	241	20.4	79.6	0.0	1.44E+05	601	8.19
123	241	20.0	80.0	0.0	1.44E+05	599	8.30
124	230	20.0	80.0	0.0	1.44E+05	599	5.15
125	230	20.0	80.0	0.0	1.44E+05	599	5.49
126	250	20.0	80.0	0.0	1.44E+05	599	12.64
127	250	19.6	80.4	0.0	1.44E+05	600	12.77
128	250	19.3	80.7	0.0	1.44E+05	600	12.86
129	250	19.6	80.4	0.0	1.44E+05	600	12.63
130	250	20.0	80.0	0.0	1.44E+05	599	12.35
131	250	20.4	79.6	0.0	1.44E+05	601	12.00
132	250	20.8	79.2	0.0	1.44E+05	600	11.83
133	250	20.4	79.6	0.0	1.44E+05	600	11.91
134	250	19.9	80.1	0.0	1.47E+05	611	12.11
135	250	20.0	80.0	0.0	2.16E+05	900	8.14
136	250	19.6	80.4	0.0	2.17E+05	902	8.26
137	250	19.1	80.9	0.0	2.16E+05	902	8.36
138	250	19.6	80.4	0.0	2.16E+05	902	8.23
139	250	20.0	80.0	0.0	2.16E+05	900	8.12
140	250	20.4	79.6	0.0	2.16E+05	899	7.97
141	250	20.8	79.2	0.0	2.16E+05	899	7.82
142	250	20.3	79.7	0.0	2.16E+05	899	7.96
143	250	20.0	80.0	0.0	2.16E+05	900	8.05
144	230	20.0	80.0	0.0	1.44E+05	599	5.14
145	230	20.0	80.0	0.0	1.44E+05	599	5.23
146	261	19.9	80.1	0.0	2.16E+05	900	12.94
147	261	19.6	80.4	0.0	2.17E+05	902	13.05
148	261	19.2	80.8	0.0	2.16E+05	902	13.22
149	261	19.6	80.4	0.0	2.16E+05	902	13.07
150	261	20.0	80.0	0.0	2.16E+05	900	12.74
151	261	20.4	79.6	0.0	2.16E+05	899	12.66

Test #	Temperature [°C]	CO_2 [%]	H_2 [%]	Ar [%]	GHSV [h-1]	Flowrate [NmL/min]	CH_4 yield [%]
152	261	20.8	79.2	0.0	2.16E+05	900	12.45
153	260	20.3	79.7	0.0	2.16E+05	899	12.50
154	260	20.0	80.0	0.0	2.16E+05	900	12.66
155	271	20.0	80.0	0.0	2.16E+05	900	18.79
156	271	19.6	80.4	0.0	2.16E+05	902	18.95
157	271	19.2	80.8	0.0	2.16E+05	902	19.40
158	271	19.6	80.4	0.0	2.17E+05	902	19.14
159	271	20.0	80.0	0.0	2.16E+05	900	18.91
160	271	20.4	79.6	0.0	2.16E+05	899	18.65
161	271	20.8	79.2	0.0	2.16E+05	899	18.37
162	271	20.3	79.7	0.0	2.16E+05	899	18.63
163	271	20.0	80.0	0.0	2.16E+05	900	18.85
164	231	20.0	80.0	0.0	1.44E+05	599	5.31
165	251	14.2	57.2	28.5	2.16E+05	899	10.90
166	251	14.2	71.5	14.3	2.16E+05	898	12.13
167	250	14.2	85.8	0.0	2.16E+05	899	11.73
168	251	14.0	42.4	43.6	2.18E+05	909	10.92
169	250	13.9	27.8	58.3	2.21E+05	922	9.73
170	250	5.6	33.3	61.1	2.16E+05	900	20.48
171	251	8.3	33.3	58.4	2.16E+05	898	15.43
172	251	6.7	33.3	59.9	2.16E+05	900	17.54
173	251	11.2	33.7	55.1	2.16E+05	900	12.50
174	251	18.2	33.3	48.5	2.16E+05	900	8.17
175	251	33.3	33.4	33.3	2.14E+05	893	4.66
176	251	66.7	33.3	0.0	2.16E+05	900	2.38
177	251	14.2	14.3	71.5	2.16E+05	899	7.05
178	249	14.2	7.2	78.6	2.16E+05	900	4.47
179	230	19.9	80.1	0.0	1.44E+05	599	5.29
180	261	14.2	85.8	0.0	2.16E+05	899	16.55
181	261	14.2	71.5	14.3	2.16E+05	899	16.21
182	261	14.2	57.2	28.6	2.16E+05	900	15.92
183	261	14.2	42.9	42.9	2.16E+05	899	15.50
184	261	14.2	28.5	57.3	2.16E+05	899	13.68
185	260	14.2	21.4	64.4	2.16E+05	900	12.36
186	259	14.2	14.3	71.5	2.16E+05	900	10.26
187	259	14.2	10.7	75.0	2.16E+05	900	8.42
188	259	14.2	7.1	78.7	2.16E+05	898	6.37
189	260	6.7	33.3	60.0	2.16E+05	900	25.83
190	261	8.3	33.3	58.3	2.16E+05	899	22.56
191	262	11.2	33.6	55.2	2.14E+05	892	18.30
192	262	16.6	33.4	50.0	2.16E+05	899	13.50
193	260	22.2	33.4	44.4	2.16E+05	900	9.68
194	261	33.3	33.3	33.3	2.16E+05	899	6.80
195	261	42.7	33.3	24.0	2.16E+05	900	5.40
196	261	66.7	33.3	0.0	2.16E+05	900	3.50
197	230	20.0	80.0	0.0	1.44E+05	599	5.20
198	239	14.2	85.8	0.0	2.16E+05	899	6.93
199	240	14.2	71.5	14.3	2.16E+05	899	6.88
200	240	14.2	57.2	28.5	2.16E+05	900	6.72
201	240	14.2	42.9	42.9	2.16E+05	899	6.59
202	240	14.2	28.6	57.2	2.16E+05	899	6.06

**Test #**	**Temperature [°C]**	**CO_2 [%]**	**H_2 [%]**	**Ar [%]**	**GHSV [h-1]**	**Flowrate [NmL/min]**	**CH_4 yield [%]**
203	240	14.2	21.5	64.3	2.16E+05	900	5.42
204	240	14.2	14.3	71.5	2.16E+05	900	4.63
205	240	14.2	10.7	75.1	2.16E+05	900	3.98
206	240	14.3	7.1	78.6	2.16E+05	899	3.19
207	240	5.6	33.4	61.1	2.16E+05	900	13.03
208	240	6.7	33.3	60.0	2.16E+05	900	11.29
209	240	8.3	33.4	58.3	2.16E+05	900	9.52
210	240	11.2	33.8	55.0	2.14E+05	892	7.81
211	240	16.7	33.3	50.0	2.16E+05	899	5.29
212	240	22.2	33.3	44.4	2.16E+05	900	4.02
213	240	33.3	33.4	33.3	2.16E+05	899	2.85
214	240	42.8	33.4	23.9	2.16E+05	900	2.23
215	240	66.7	33.3	0.0	2.16E+05	900	1.47
216	230	20.0	80.0	0.0	1.44E+05	599	4.85
